# Fracture Load of Mesio–Occluso–Distal Composite Restorations Performed with Different Reinforcement Techniques: An In Vitro Study

**DOI:** 10.3390/polym15061358

**Published:** 2023-03-08

**Authors:** Nassreen Albar, Waad Khayat

**Affiliations:** 1 Department of Restorative Dentistry, College of Dentistry, Jazan University, Jazan 45142, Saudi Arabia; 2 Department of Restorative Dentistry, College of Dentistry, Umm Al-Qura University, Makkah 24381, Saudi Arabia

**Keywords:** mesio–occluso–distal restoration, composite, reinforced composite, polyethylene, Ribbond

## Abstract

Background: Mesio–occluso–distal (MOD) cavity preparations are often fragile due to the amount of tooth and carious structure removed. MOD cavities can often fracture if left unsupported. Aim: The study investigated the maximum fracture load of mesi–occluso–distal cavities restored using direct composite resin restorations with various reinforcement techniques. Method: Seventy-two freshly extracted, intact human posterior teeth were disinfected, checked, and prepared according to predetermined standards for mesio–occluso–distal cavity design (MOD). The teeth were assigned randomly into six groups. The first group was the control group restored conventionally with a nanohybrid composite resin (Group I). The other five groups were restored with a nanohybrid composite resin reinforced with different techniques: the ACTIVA BioACTIVE-Restorative and -Liner as a dentin substitute and layered with a nanohybrid composite (Group II); the everX Posterior composite resin layered with a nanohybrid composite (Group III); polyethylene fibers called “Ribbond” placed on both axial walls and the floor of the cavity, and layered with a nanohybrid composite (Group IV); polyethylene fibers placed on both axial walls and the floor of the cavity, and layered with the ACTIVA BioACTIVE-Restorative and -Liner as a dentin substitute and nanohybrid composite (Group V); and polyethylene fibers placed on both axial walls and the floor of the cavity and layered with the everX posterior composite resin and nanohybrid composite (Group VI). All teeth were subjected to thermocycling to simulate the oral environment. The maximum load was measured using a universal testing machine. Results: The highest maximum load was exhibited by Group III with the everX posterior composite resin, followed by Group IV, Group VI, Group I, Group II, and Group V. A statistically significant difference was demonstrated between groups (*p* = 0.0023). When adjusting for multiple comparisons, there were statistical differences specific to comparisons between Group III versus I, Group III versus II, Group IV versus II, and Group V versus III. Conclusions: Within the limitations of the current study, it can be concluded that a higher maximum load resistance can be achieved (statistically significant) when reinforcing nanohybrid composite resin MOD restorations with everX Posterior.

## 1. Introduction

Dental caries is the most common oral disease in the world. Conservative estimates indicate that over 2.5 billion people suffer from dental caries [1]. This has led to a sharp rise in demand for tooth-colored resin composites for restorations including for posterior teeth. Advances in dental biomaterials and improved cavity design have made tooth-colored restorations the default approach in modern caries management. Composite restorations have been shown to have excellent mechanical and biological properties, with an overall low failure rate [2].

Inconsistent and infrequent dental visiting patterns, dental neglect, and secondary caries can result in large carious defects, and subsequently, may require extensive cavity preparation [3,4]. Tooth preparation and carious tissue removal, followed by restoration, are pillars of caries treatment. Tooth preparation creates the superstructure foundation for receiving the restorative material. Successive restorations tend to increase in size, increasing the risk of subsequent tooth fractures and a shorter life span of the restoration [5].

Cavities that require extensive preparation experience an enormous loss of hard dental tissues, which proportionally decreases the strength of the teeth and results in fractures [6]. Preparation involving the mesial, occlusal, and distal surfaces of a tooth are known as mesio–occluso–distal (MOD) cavities. MOD cavity designs can result in up to a 63% loss of cuspal stiffness, weakening the tooth [7]. MOD designs are prone to fracture, especially in the posterior teeth, regardless of the thickness of the remaining walls [8]. The accumulated tooth loss and fragility can be countered by reinforcing the remaining tooth structure. Reducing the interfacial stress concentrations in large composite restorations can reduce cuspal deflection [9,10].

The longevity of restorations is affected by the age and properties of the restorative material. Materials that adhere to dentin and act as a base can ensure uniform stress distribution [11]. Problems of fragility are compounded by polymerization shrinkage and related stress, which contribute to the imperfect marginal adaptation [12]. However, fracture strength continues to be one of the decisive characteristics in successful clinical outcomes. Resistance to fracture is a measure of fatigue resistance (i.e., tolerance to damage), which may be indicative of structural performance and longevity [13].

Throughout the years, different methods have been used to reinforce restorations, either by manipulating the composition of the materials, amount, and size of fillers, or by using the recent approach of placing fibers under the restoration as a form of reinforcement [14]. Polymer composites are the first material of choice to solve most dental pathologies such as caries and trauma in today’s practice. Composites earned their popularity due to their superior properties such as biocompatibility, excellent esthetics, and antibacterial features. These polymer fiber-reinforced materials were developed to resolve the shortcomings of bulk materials including a lack of adequate toughness and strength for load bearing. Fiber-reinforced composites provide rigidity and increase the fracture resistance by fortifying an unsupported tooth structure. Short-fiber-reinforced composites have been shown to resist crack propagation compared to conventional composites while demonstrating good physical, mechanical, and thermal properties [15,16].

Several types of resin-reinforced materials that do not affect the esthetic properties of the restoration are available. The mechanical properties of the material are influenced by the orientation and quantity of the fibers, along with the characteristics of the polymer matrix they are embedded within. Reinforcing fibers can be arranged unidirectionally or bidirectionally (weave) or in a continuous random orientation (mat). Creating a layer of ultra-high-molecular-weight polyethylene fiber laid down in a leno weave pattern has been shown to increase the fracture resistance of endodontically treated teeth [8].

Commercially available since 1992, Ribbond (Ribbond Inc., Seattle, WA, USA) is composed of leno-woven polyethylene fibers that allow for the adaptation of the restorative material close to the contours of the tooth [17]. The composite resin mechanically interlocks into different planes of the three-dimensional structure and reduces the propagation of microcracks during polymerization [18].

The everX Posterior composite (GC Corp, Tokyo, Japan) incorporates short E-glass fiber fillers, with lengths ranging from 0.6 to 1.5 mm (mainly, 0.8 mm) [19], and inorganic particulate fillers into a cross-linked polymer matrix called the semi-interpenetrating polymer network (IPN). The random short-fiber orientation and the semi-IPN allows for better bonding and improves the toughness of the composite [20,21]. The fiber layer acts like a dentin substitute to ultimately improve the load-bearing capacity of the restoration [22].

ACTIVA (Pulpdent Corporation, Watertown, MA, USA) is a material recently introduced into the dental market that combines the strength and esthetics of composites with all the benefits of glass ionomers and has a wide range of restorative applications including complex multiple surface restorations. Patented bioactive ionic resin, patented rubberized resin, and bioactive ionomer glass are the key components of this material that mimic the physical and chemical properties of natural teeth due to its high release and recharge of calcium, phosphate, and fluoride ions, and which also add to its toughness and durability [23,24].

Few published studies have focused on comparing the various modern reinforcement techniques with conventional composite restorations [8,13,22,25]. To date, there has been little research directly examining the maximum fracture load of MOD cavity preparations restored with different reinforced fiber resins. The effect of these techniques on the maximum fracture load remains unclear. This study aimed to evaluate the maximum fracture load of mesio–occluso–distal direct composite resin restorations (restored with different reinforcement techniques) compared to teeth restored conventionally.

## 2. Materials and Methods

In the present in vitro study, seventy-two intact human molars extracted for periodontal or orthodontic causes were used after obtaining informed consent from patients. This study adhered to the Declaration of Helsinki and ethical approval was obtained from the Institutional Review Board (IRB) of Jazan University, REC-43/11/267.

### 2.1. Sample Size Calculation

A sample size calculation was conducted using G Power (Version 3.1) (Faul, Erdfelder, Lang, and Buchner 2007, Heinrich-Heine-Universität Düsseldorf, Germany). A sample size of *n* = 12 per group was adequate to obtain a Type I error rate of 5 percent and a power of 80 percent with an effect size of 0.5.

### 2.2. Specimen Preparation

The freshly extracted teeth were cleaned to remove any periodontal soft tissue or calculus from the root and crown using a Gracey hand scaler (Hu-Friedy, Chicago, IL, USA) and disinfected via immersion in a 0.5% sodium hypochlorite solution for 15 min. Sterilized teeth were stored in distilled water until use. The teeth were determined to be intact with no extensive wear, caries, cracks, fractures, or previous restorations through visual inspection. Additionally, the selected teeth were chosen to be of comparable buccolingual and mesiodistal dimensions and morphology. Teeth were fixed to a resin block at a level of 2 mm apical to the cementoenamel junction.

### 2.3. Cavity Preparation

**Figure 1:**
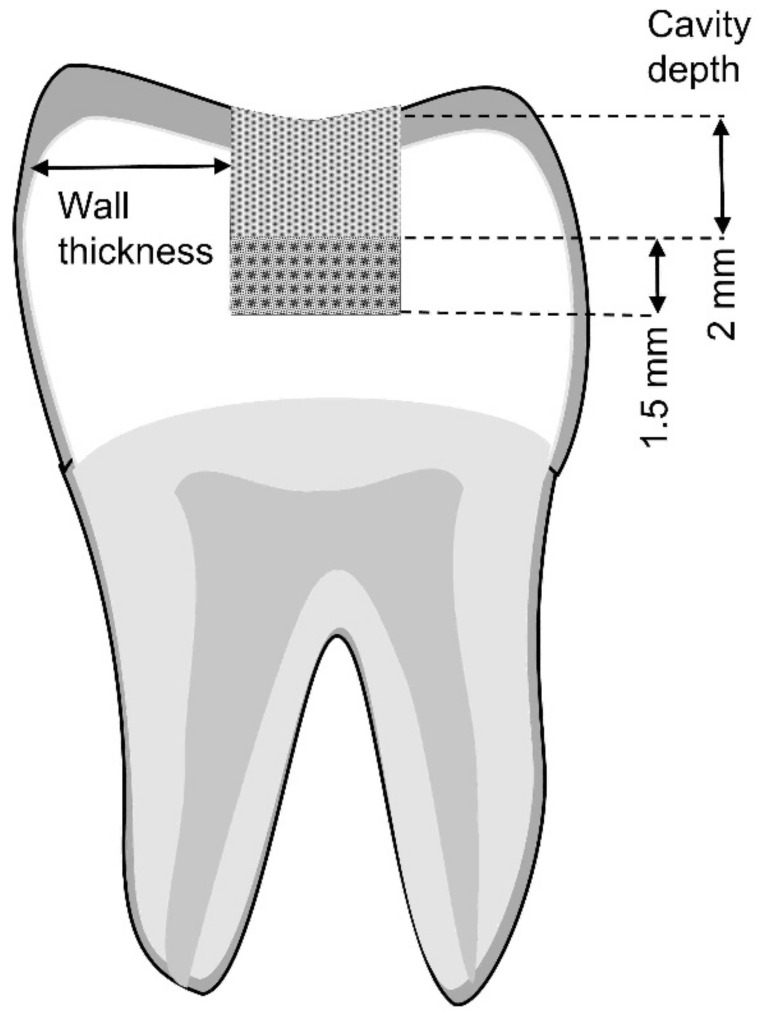
The author’s schematic faciolingual cross-section of the cavity depth (occlusal and proximal) prepared in molars subjected to the maximum fracture load testing.

We followed the standard MOD cavity preparation, as previously described by Forster et al., by using a round-end parallel diamond bur (Komet CE) [26]. The cavities were prepared using a high-speed handpiece and water coolant by the same dentist on all teeth. The bur was initially positioned in the midline of the occlusal surface of the tooth. The cavity walls were prepared parallel to the axis of the tooth. The thickness of the opposing walls at the cavity base was continuously monitored throughout the preparation by using a digital caliper (Mitutoyo Corp., Kawasaki, Japan). The occlusal cavity had a depth of 2 mm, which was measured using a periodontal probe (Hu-Friedy, Chicago, IL, USA), and a buccolingual (isthmus) width that was one-third of the intercuspal distance on each tooth, measured by the same operator. The facial and lingual walls were parallel, and the cavosurface angle was 90°. All the internal line angles were rounded. The width of the proximal box was one-third of the total faciolingual distance of the marginal ridge. The axial depth of the proximal cavity was 1.5 mm, and the gingival floor was seated at 1 mm coronal to the cementoenamel junction with margins on the enamel [27] (Figure 1). A water-resistant pencil was used to draw the extension of the cavity outline on the predetermined extension.

**Figure 2:**
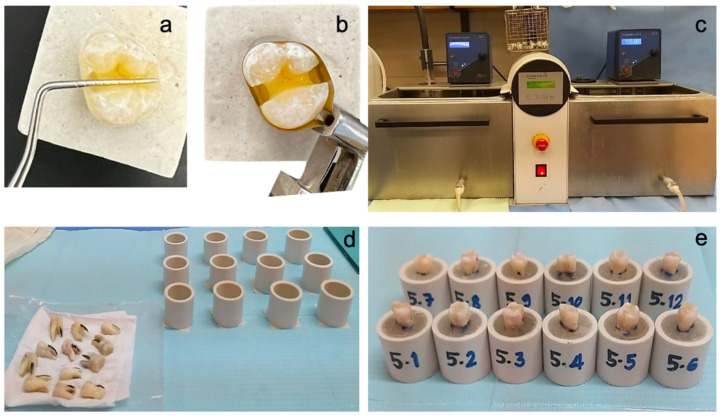
(**a**) Measurements of the cavities were taken for standardization purposes. (**b**) The matrix band placed around the cavity preparation to restore the proximal walls before adding the designated restorations to each group. (**c**) The restored teeth were placed in the thermocycling machine set at 10,000 cycles. (**d**) The aged teeth were mounted in special molds made specific for the Instron placement. (**e**) The molds were labeled according to group number (first digit) and sample number (second digit).

In total, 72 molars were randomly distributed between six groups using R statistical software (version 3.1.2, R Foundation for Statistical Computing, Vienna, Austria) (Figure 2). Following group allocation, each specimen was labeled on the back by using a permanent pen with a letter indicating the assigned group (A–F) and a number (1–12), indicating the number of the specimen within the group.

**Figure 3:**
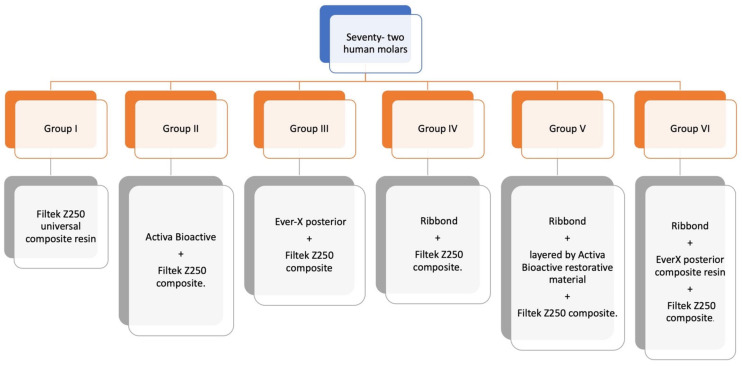
Distribution of the six groups for maximum load testing.

- Group I was the control group restored conventionally with the Nanohybrid Filtek Z250 universal composite resin (3M ESPE), using a simple layering technique.
- Group II was restored using the ACTIVA BioACTIVE-Restorative material (Pulpdent Corporation) and liner as a dentin substitute, which was layered with the Nanohybrid Filtek Z250 universal composite (3M ESPE).
- Group III was restored with the everX Posterior composite resin (GC Corporation), which was layered with the Nanohybrid Filtek Z250 universal composite (3M ESPE).
- Group IV was restored using polyethylene fibers (Ribbond, Inc.) placed on both axial walls and the cavity floor, layered with the Nanohybrid Filtek Z250 universal composite (3M ESPE).
- Group V was restored with polyethylene fibers (Ribbond, Inc.) placed on both axial walls and the floor of the cavity, layered with the ACTIVA BioACTIVE-Restorative material (Pulpdent Corporation) and liner as a dentin substitute and the Nanohybrid Filtek Z250 universal composite (3M ESPE).
- Group VI was restored with polyethylene fibers (Ribbond, Inc.) placed on both axial walls and the floor of the cavity, layered with the everX Posterior composite resin (GC Corporation) and the Nanohybrid Filtek Z250 universal composite (3M ESPE) (Figure 3). Composition of each material is discussed in Table 1 below.

Group I was the control group. All cavities belonging to the control group were restored conventionally using the Nanohybrid Filtek Z250 universal composite resin, following the manufacturing instructions for bonding (Ambar, FGM Dental Group), the incremental layering of restorations, and curing.

The teeth in the experimental groups (II, III, IV, V, and VI) were restored with different techniques according to the group they were assigned to. Group II was restored using ACTIVA BioACTIVE-Restorative and liner as a dentin substitute, which was layered with the Nanohybrid Filtek Z250 universal composite resin. The bonding protocol was applied according to the manufacturer’s instructions. A 0.5 mm layer of BioACTIVE-Liner was applied on the floor, followed by a layer of the ACTIVA BioACTIVE material to replace the dentin and a layer of the Nanohybrid Filtek Z250 universal composite resin to finish the restoration. Group III was restored with the everX Posterior composite resin in place of dentin, which was layered with the Nanohybrid Filtek Z250 universal composite resin. Group IV was restored with the polyethylene fibers called “Ribbond” (Ribbond Inc., Seattle, WA, USA), which were placed on both axial walls and the cavity floor, layered with the Filtek Z250 universal composite resin. The bonding protocol was applied according to the manufacturer’s instructions. Then, a 0.5 mm layer of a flowable composite resin (Opallis Flow, FGM Dental Group) was placed into the cavity. A small piece of the fiber was wetted with an unfilled bonding resin and embedded into the composite resin layer (0.5 mm away from the external margin of the tooth structure). The fiber was placed on the floor of the cavity and axial walls. The last layer was placed and cured as per the recommended instructions. Group V was restored with polyethylene fibers placed on both axial walls and the floor of the cavity, and layered with the BioACTIVE-Liner and -Restorative as a dentin substitute and with the Nanohybrid Filtek Z250 universal composite resin to finish the restoration. Group VI was restored with polyethylene fibers (Ribbond, Inc.) placed on both axial walls and the floor of the cavity, and layered with the everX Posterior composite resin (GC Corporation) and the Nanohybrid Filtek Z250 universal composite resin.

All of the specimens were finished and polished according to the manufacturer’s instructions, and then stored in distilled water at a temperature of 37 °C.

To simulate the functioning in the oral cavity, teeth were subjected to thermocycling (SD Mechatronik Thermocycler 1100- Feldkirchen-Westerham, Germany) for 10,000 cycles at 5 °C and 55 °C. Each cycle corresponded to a 15 s bath at each temperature and a transfer time of 5 s (ISO Recommendations 11405:1994) [31].

### 2.4. Evaluation of Maximum Load

**Figure 4:**
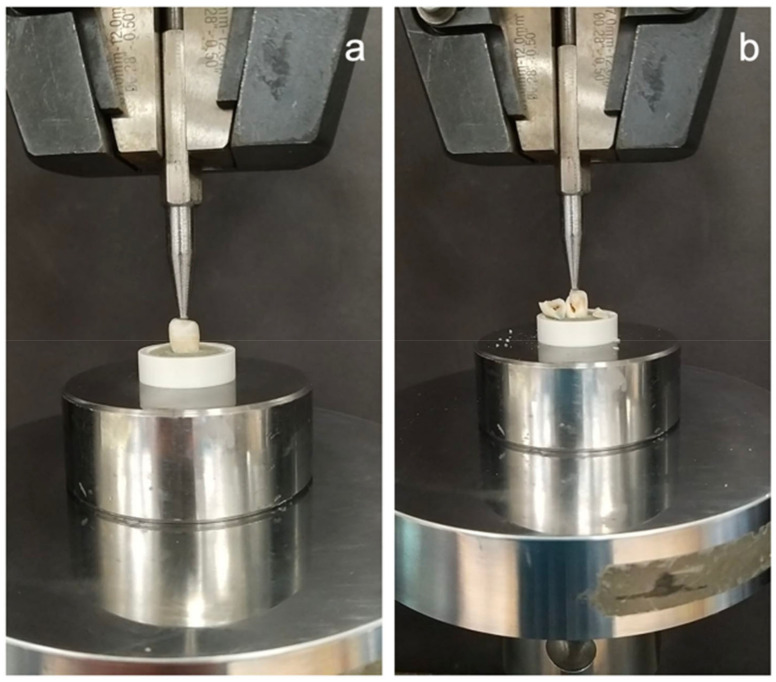
(**a**) The specimen was subjected to an axial compressive load meant to cause fracturing by using a 3 mm diameter steel ball that contacts the restoration at the center area at a crosshead speed of 0.5 mm/min. (**b**) Fractured specimen.

All specimens were tested for the maximum fracture load resistance within 24 h after thermocycling. The specimens were evaluated using a universal testing machine (Instron 5944, Instron Corp., Norwood, MA, USA). Each specimen was mounted using a cylindrical shaped fixture to hold the tooth. The center of each tooth was determined based on the anatomy and the measurements of the restoration and marked with a permanent marker. The samples were subjected to an axial compressive load by using a 3 mm diameter steel ball that contacts the restoration at the marked center at a crosshead speed of 0.5 mm/min. The maximum load (Newtons) was recorded when the fracture occurred. The settings were controlled, and the results were displayed using BlueHill software 3.22.1373, Norwood, MA, USA (Figure 4).

**Figure 5:**
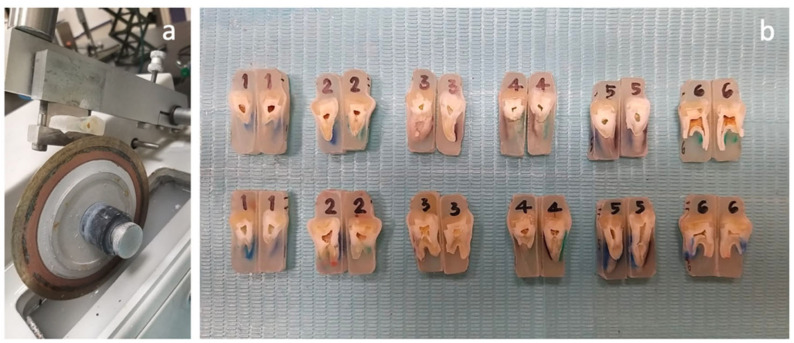
(**a**) Specimens were sectioned and prepared for scanning electron microscopy (SEM). (**b**) Labelling the samples according to the designated group (digit placed above).

**Figure 6:**
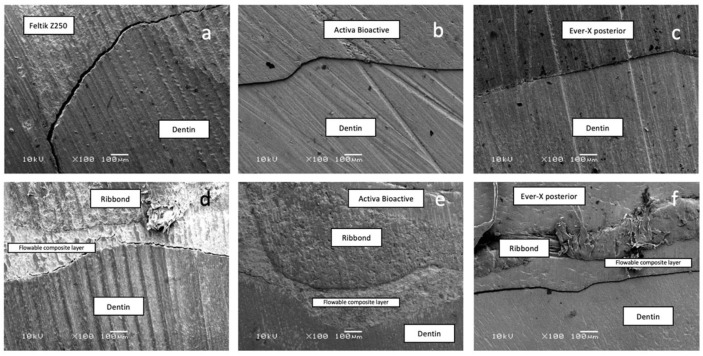
(**a**) Scanning electron image of Group 1. (**b**) Group 2. (**c**) Group 3. (**d**) Group 4. (**e**) Group 5. (**f**) Group 6.

Two extra specimens from each group were prepared for the microstructure analysis using a scanning electron microscope (Field Emission Variable Pressure Analytic Scanning Electron Microscope FESEM-VP-Hitachi SU6600 with Oxford Instrument AZtec X-Max 50 SDD Energy Dispersive Spectrometer, Hitachi High Tech, Oxford Instruments, Abingdon, UK). Each specimen was embedded into resin, sectioned, and sonicated with ethanol (Fisher Scientific, Pittsburgh, PA, USA) to clean it, then dried in an oven (Vacuum oven1410, VWR, Radnor, PA, USA) at 90 °C for 30 min. The microstructure of specimens was examined with a voltage of 10 kV at magnifications of 100× (Figure 5 and Figure 6)

### 2.5. Statistical Analysis

The primary outcome was the maximum load. Medians with interquartile range were reported for descriptive statistics. Normality was initially assessed visually using a scatterplot. Visual inspection indicated skewed distributions, which in addition to the small sample sizes, were conferred using a non-parametric test (i.e., Kruskal–Wallis test). If the overall test was significant, we performed comparisons using the pairwise Wilcoxon rank-sum test with *p*-value corrections by Benjamini and Hochberg to determine which groups were statistically different. All *p*-values less than 0.05 were considered statistically significant. Data were organized using Excel (Microsoft 365). Analyses were performed by using SPSS software (version 23, SPSS Inc., Chicago, IL, USA) and R (version 3.1.2, R Foundation for Statistical Computing, Vienna, Austria).

## 3. Results

**Figure 7:**
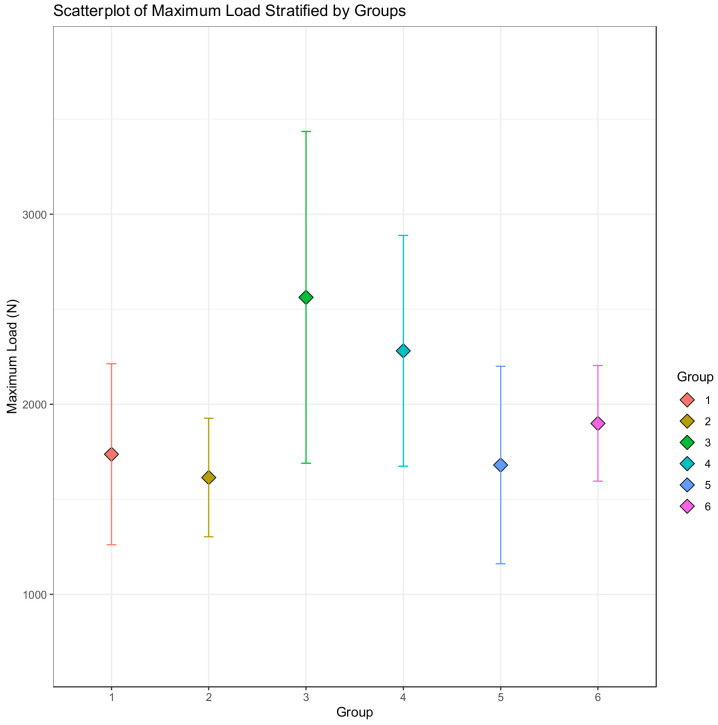
Scatterplot depicting the distributions of data for the maximum load measured in Newtons. Diamonds indicate the means and error bars represent one standard deviation. Visual inspection suggests non-normally distributed data.

A scatterplot depicting the distributions of the maximum load is shown in Figure 7.

The highest median maximum load was exhibited by Group III (median 2693.1 [IQR 2078.6 to 3077.5] MPa), followed by Group IV (2436.0 [1689.5 to 2752.3] MPa), Group VI (1869.2 [1763.8 to 2193.6]), Group I (1643.1 [1387.4 to 2057.7]), Group II (1610.9 [1444.5 to 1871.1]), and finally, Group V (1550.4 [1274.6 to 2152.0]). The Kruskal–Wallis showed a statistically significant difference between the groups with respect to the maximum load (*p* = 0.0023). Pairwise group comparisons with *p*-value corrections indicated that there were significant differences between Group III versus Group I (*p* = 0.038), Group III versus Group II (*p* = 0.017), Group IV versus Group II (*p* = 0.038), and Group V versus Group III (*p* = 0.038). Table 2 shows the corresponding medians with interquartile ranges for each group for the outcome of maximum load (Table 2).

## 4. Discussion

A decisive factor for determining the fracture resistance of the restoration is the amount of tooth structure left after cavity preparation [32]. A major contributing factor leading to tooth fragility is the extensive cavity preparation that causes fractures in the cusps (partial or complete) or the roots of posterior teeth [11]. When compared to nonprepared teeth, MOD cavity preparations cause a 54% reduction in fracture resistance [32]. Several strategies have been adopted to overcome this disadvantage [33]. Different restorative materials have been introduced to enhance the strength of the remaining tooth structure and improve the properties of conventional materials. Suitable material selection compensating for the lost tooth structure and that supports the remaining dental tissue is fundamental to achieve a successful treatment [34]. Different types of fiber-reinforced composites are among the most important examples of modified composites due to their tooth strengthening effects [35]. This study aimed to evaluate the maximum fracture load of MOD direct composite resin restorations in teeth that were restored with different reinforcement techniques and compare them with teeth restored conventionally.

In the present study, it was observed that the highest fracture load was shown by Group III (teeth restored with everX Posterior and layered with a nanohybrid composite resin). The everX composite resin material is composed of short E-glass fibers randomly oriented in a resin matrix (bis-GMA and TEGDMA cross-linked monomers) to reinforce the material [36,37]. The short, multidirectional glass fibers in the resin composite and the locked interwoven series of polyethylene fibers on the interfacial stresses create a multitude of load paths, improving the fracture resistance [36,38]. When Ribbond fibers were placed beneath everX Posterior in Group VI, the fracture load was reduced compared to Group III, though the difference between the results was not statistically significant. As explained by Vallittu [39], specimens examined using a scanning electron microscope demonstrated a relatively poor fiber–matrix coupling. The adhesion of the resin composite to polyethylene fibers might be negatively affected because of the difficulty in regard to the impregnation of the polyethylene fibers, resulting in the restoration having less favorable properties [40]. In other words, heterogenic combining the two different materials may negatively influence the results because of the difficulty experienced in the adhesion between Ribbond and everX Posterior.

The second highest median fracture load value was exhibited by Group IV (restored with Ribbond polyethylene fibers and layered with a nanohybrid composite resin). The reinforcement of nanohybrid composite resin restorations with Ribbond improved the fracture load in comparison to the control group (Group I); however, the statistical difference was not significant between them. These results were supported by findings from previous studies [41,42]. Ribbond fibers have a lock-stitch pattern that transfers the forces through the fibrous weave without propagating the stress into the resin [43]. In addition, fibers placed on opposing walls may act as an internal splint, acting as a buffer to absorb stress and redirecting or preventing crack formation and propagation to enhance the mechanical behavior and the fracture resistance of the material [38].

In our study, Group IV presented a comparable fracture strength to Group III in MOD restorations (with no statistically significant difference between these groups). In agreement with our results, a previous study that evaluated similar materials used for core build-up in endodontically treated molars also reported no statistically significant difference in the fracture resistance of everX composite resin restorations compared to Ribbond reinforced restorations [44]. On the other hand, there was a statistically significant difference between the fracture resistance of everX Posterior composite restorations in comparison with the Ribbond restorations [38]. The manual impregnation of Ribbond fibers can be inappropriately performed, which may create voids in the matrix, and consequently, could lead to the premature failure of the restoration; this is in contrast to everX Posterior, in which fibers are incorporated during manufacturing [45]. From a clinical perspective, everX Posterior is easier to apply, exhibits less technique sensitivity, and is less time consuming than Ribbond, which are advantages in daily dental practice.

In the present study, the lowest median fracture load values were reported by Groups II and V. The ACTIVA BioACTIVE material was reported to possess the properties of a resin-modified glass-ionomer cement, in addition to having a modified resin matrix that improved the resilience of the material and enhanced its physical properties in small to moderate restorations [46]. The placement of Ribbond fibers underneath ACTIVA BioACTIVE did not improve the fracture resistance of the MOD restoration, which might be explained by the negative influence of combining the two different materials due to weak adhesion between Ribbond and ACTIVA BioACTIVE. The SEM image (Figure 6) presents an idea about the pattern of interface between the materials.

This in vitro study has achieved its purpose; however, there were some limitations to the in vitro studies, conducted under static loading conditions, which vary from clinical situations with dynamic loading conditions. Such studies only provide limited, but valuable, preliminary data as a guide for further clinical investigations to evaluate the clinical performance of the materials. Moreover, it is quite inequitable to compare the fracture load data from the laboratory studies due to different variables such as the morphology of the cavity designs (class II vs MOD), the procedures of applying the restorations and the different study protocols, the influence of different reinforcing bonded materials to reinforce the restored tooth, the challenges of computing the fracture load accurately due to the complex geometry of the tooth structure, and location and distribution of the applied forces during the test, for example, the application of load represented by the piston in the Instron machine in the center of the restoration will result in different outcomes than when placed in the isthmus area, which is known to be a weak area of the restoration. Future studies are needed to compare and study the influence of these variables and its correlations to the outcome. Furthermore, further studies should investigate the bonding of various materials and the pattern of failure (adhesive, cohesive or combined). The SEM analysis of fractured specimens is also a scope of interest for future studies.

## 5. Conclusions

Within the limitations of the current study, it can be concluded that a higher maximum load resistance can be achieved when reinforcing nanohybrid composite resin MOD restorations with everX Posterior or Ribbond. We specifically identified that everX Posterior had a statistically significant higher maximum load resistance versus the control and versus the polyethylene fibers layered with ACTIVA BioACTIVE-restorative material. Both everX Posterior and the polyethylene fiber layered nanohybrid Filtek had a higher maximum load resistance versus the ACTIVA BioACTIVE-restorative material. Further studies are required to confirm and validate these findings.

## Figures and Tables

**Table 1:** Composition of the matrix of polymer materials used in the study.

Polymer	Composition
**Filtek Z250**	BIS-GMA (Bisphenol A dislvcidy] ether dimethacrylate), UDMA (ure-thane dimethacrylate), and Bis-EMA (bisphenol A polyethylene glycol diether dimethacrylate) [28]
**ACTIVA BioACTIVE-Restorative material**	Blend of diurethane and other methacrylates with modified polyacrylic acid [29]
**everX Posterior**	(1-methylethylidene) bis [4,1-phenyleneoxy(2-hydroxy-3,1-propanediy!)] bismethacrylate. 2,2’-ethylenedioxy diethyl dimethacrylate, dipheny| (2,4,6-trimethylbenzoyl)phosphine oxide, 6-tert-butyl-2,4-xylenol [30]

**Table 2:** Comparison of the maximum load between the groups.

Outcome	Group	*n*	Median	IQR	*p*-Value
Maximum load (Newtons)	Group I	12	1643.1	1387.4 to 2057.7	0.0023
	Group II	12	1610.9	1444.5 to 1871.1	
	Group III	12	2693.1	2078.6 to 3077.5	
	Group IV	12	2436.0	1689.5 to 2752.3	
	Group V	12	1550.4	1274.6 to 2152.0	
	Group VI	12	1869.2	1763.8 to 2193.6	

Statistical significance set at 0.05; *n*: number of samples per group; IQR: interquartile range. Kruskal–Wallis statistical analysis was performed.

## Data Availability

All of the data underlying the results of this current study are available as part of the article. No additional sources or data were required.
